# What implies registered nurses’ leadership close to older adults in municipal home health care? A systematic review

**DOI:** 10.1186/s12912-020-00413-1

**Published:** 2020-04-20

**Authors:** Maria Claesson, Lise-Lotte Jonasson, Elisabeth Lindberg, Karin Josefsson

**Affiliations:** 1 Faculty of Caring Science, Work Life and Social Welfare, University of Borås, 501 90 Borås, Sweden; 2 The Faculty of Health, Science and Technology, Karlstad University, Karlstad, 651 88 Sweden

**Keywords:** Home health care, Leadership, Multi-artist, Municipal, Older adults, Registered nurse, Systematic review

## Abstract

**Background:**

Registered nurses are key figures in municipal home health care for older adults. Thus, registered nurses’ leadership is crucial to a successful and preventive care process as well as a supportive organization in order to achieve safe care. However, there is limited research on what registered nurses’ leadership implies close to older adults in municipal home health care. Thus, the aim is to compile and critically evaluate how international research results describe registered nurses’ leadership close to older adults in municipal home health care.

**Methods:**

A systematic literature review was performed in accordance with a qualitative research study. The main search was conducted on 20 April 2018. The review was reported according to the PRISMA guidelines and is registered in the PROSPERO database (ID# CRD42019109206). Nine articles from PubMed and CINAHL meet the quality criteria. A synthesis of data was performed in four stages according to qualitative research synthesis.

**Results:**

Ten themes describe what registered nurses’ leadership close to older adults in municipal home health care entails: trust and control; continuous learning; competence through knowledge and ability; nursing responsibility on an organizational level; application of skills; awareness of the individual’s needs and wholeness; mutual support; mutual relationships; collaborating on organizational and interpersonal levels; and exposure to challenges.

**Conclusions:**

Registered nurses leading close to older adults in municipal home health care implies being multi-artists. Nursing education, including specialist education for registered nurses, should prepare individuals for their unique and complex leadership role as a multi-artist. Municipal employers require knowledge about what registered nurses’ leadership implies in order to create adequate conditions for their leadership objectives to achieve safe care. Further research is warranted to explore registered nurses’ leadership close to older adults in municipal home health care from different perspectives, such as older adults and next of kin.

## Background

Leadership is included in registered nurses’ (RNs) responsibility and competence [1]. Leadership should not be confused with management, which is a formal position that entails responsibility for operations and budget. Leadership, in contrast, seeks to produce necessary changes by developing both a vision of the future and strategies to reach that vision [1]. Nursing leadership involves ensuring that individual leaders possess the knowledge, skills and capacities required to empower those around them and to harness their strengths towards a collective effort [2]. While there are different leadership styles [3], there is no specific nursing leadership style.

RNs’ leadership in municipal home health care is based on a clear understanding of older adults’, 65 years and over, perspectives [4] in terms of meeting their needs, next of kin and care team [5]. RNs’ leadership in municipal home health care implies that they lead, prioritize, allocate, coordinate, teach and participate in nursing care with older adults, next of kin and the care team [6, 7]. Nursing leadership, clinical leadership and health care leadership are terms evident in nursing [8]. There is a need to focus on developing strong leadership in the nursing profession across the globe [9]. However, there is limited research on what RNs’ leadership implies close to older adults in municipal home health care. Therefore, the aim of this study is to compile and critically evaluate how international research results describe registered nurses’ leadership close to older adults in municipal home health care.

To meet the growing health care needs of older adults, RNs’ clinical competence and ability to make complex decisions are essential [10]. RNs are key figures in municipal home health care for older adults [1, 11] since they have the ability to put together several parts of a complex picture through their leadership, clinical competence and collaborative practice [10]. RNs in municipal home health care are responsible for older adults’ care by promoting health, preventing disease, restoring health and alleviating suffering [6]. The responsibility places high demands on competence in gerontology, geriatrics, drug management [12] and leaderhip [1]. This also includes competence to identify older adults’ life history and prevent illness as well as reduce the consequences of illness [11]. From an international perspective, there is a growing need for home health care for an upcoming aging population [13].

### Research question

What implies registered nurses’ leadership close to older adults in municipal home health care?

## Methods

### Design

This is the first study in a larger research project exploring RNs’ leadership close to older adults in municipal home health care. This systematic literature review was conducted and reported according to the PRISMA guidelines [14, 15] and is registered in the PROSPERO database (ID# CRD42019109206). The analysis was conducted according to qualitative research synthesis [16], originating from meta-ethnography [17].

### Search strategy

In collaboration with an information specialist at a university library, a search strategy was created (Table 1) between February and April 2018. The following research question was posed: What implies registered nurses’ leadership close to older adults in municipal home health care? No earlier systematic reviews were available in the subject area. One article proved useful to the design of the search strategy [18]. Two external researchers reviewed the search strategy before the main search was carried out, but the reviews did not support changes to the search strategy.

**Table 1:** Search strategy via three themes

Themes	Search terms in PubMed, 2018-04-20
Registered nurse	(((((((registered nurses [MeSH Terms]) OR community health nurses [MeSH Terms]) OR ((((((community health nurs*[Title/Abstract]) OR registered nurse*[Title/Abstract]) OR nurse*[Title/Abstract]) OR RN [Title/Abstract] OR RNs [Title/Abstract])))))
Leadership	((((leadership [MeSH Terms])) OR team nursing [MeSH Terms]) OR (((leader*[Title/Abstract])) OR team nursing [Title/Abstract])))
Home care	((community health services [MeSH Terms]) OR ((((home based [Title/Abstract]) OR home health [Title/Abstract]) OR home care [Title/Abstract] OR in home [Title/Abstract] OR at home [Title/Abstract])))
Search limiters	AND ‘last 10 years’[PDat] AND English [lang]

Test searches were performed with respect to five themes, including ‘registered nurse’, ‘leadership’, ‘home health care’, ‘older adult’ and ‘patient care’. These included MeSH terms in PubMed and CINAHL subject headings in CINAHL. Test searches using the themes ‘older adult’ and ‘patient care’ concluded that the data in these areas were severely limited. Therefore, these two themes did not contribute to the search strategy.

The main search was conducted on 20 April 2018 in the PubMed and CINAHL databases via MeSH terms in PubMed and CINAHL subject headings in combination with keywords [15] (Table 1). Thereafter, all articles that did not include the themes ‘older adult’ and ‘patient care’ were excluded from the articles’ title and abstract of the data matches. Grey literature was excluded if not falling under the category of scientific publications.

### Article selection

**Fig. 1:**
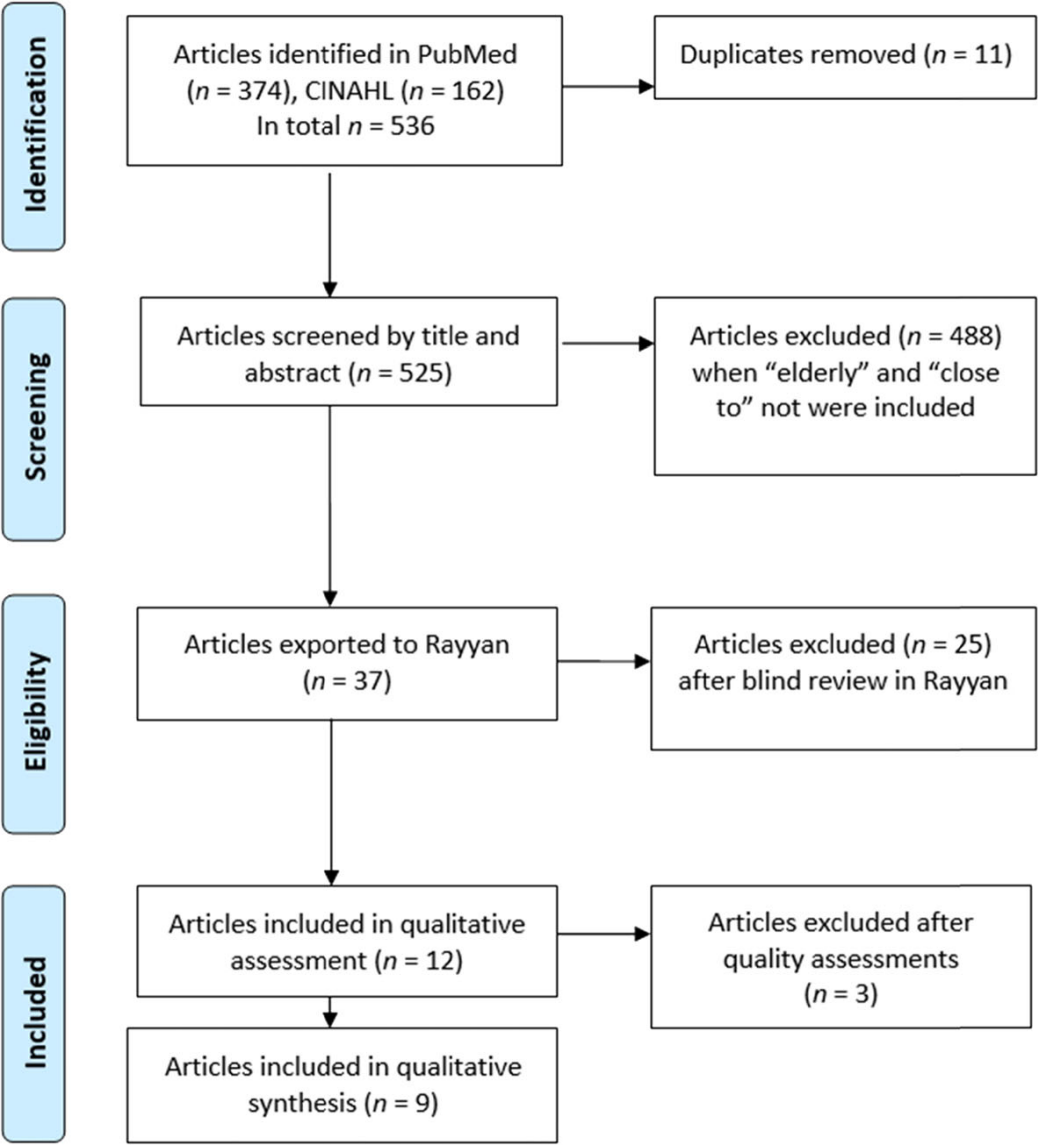
PRISMA flow chart

The article search resulted in a total of *n* = 536 articles: *n* = 374 in PubMed and *n* = 162 in CINAHL (Fig. 1). All articles were screened by title and abstract. Articles *n* = 488 did not include ‘older adults’ and ‘patient care’, and *n* = 11 duplicates were excluded. A total of *n* = 37 articles remained and were imported into Rayyan QCRI, a web-based sorting tool for systematic literature reviews [19]. Three of the authors read the titles and abstracts independently of each other and sorted the articles based on the inclusion criteria into the Rayyan QCRI. The blind articles selection in Rayyan QCRI showed a 70% consensus among the authors. The remaining articles, *n* = 12, were read again by the three authors independently of each other. There was agreement to include *n* = 12 articles in the quality assessment.

### Quality assessment

The quality assessment of *n* = 12 articles was performed by two of the authors according to a quality assessment tool to determine whether or not the quality of the articles was sufficiently good [20, 21]. The articles were read in full, and the authors completed an assessment document separately. Finally, the two authors compared their assessments with each other and *n* = 9 articles were found to have met the quality criteria.

### Characteristics of included studies

The characteristics of the included studies, *n* = 9, are described in Table 2.

**Table 2:** Characteristics of included articles with results according to the aim of current study

Authors Year Country	Design	Aim	Settings Sample	Data collection	Analysis	Results
Arnaert, A Wainwright, M (2009) Canada	Qualitative Explorative	Explore experiences, perspectives and reflection of five nurse specialists in palliative home care	Palliative home care Nurse specialists *n* = 5	Individual interviews	Content analysis	*Three major themes with subthemes* 1 Acknowledging one’s own limitations and humanness Calling for backup, learning as we go along, coping with the emotional demands and interacting with family members 2 Building a collaborative partnership Working collaboratively, sharing information, guiding home care nurses, being non-judgmental 3 teamwork and implementing palliative home care teams
Mc Garry, J (2009) UK	Qualitative Ethnographic	Explore the nature of the care relationship within the home setting between community nurses and elderly people	Home care District nurses *n* = 5 Registered nurses *n* = 9 Auxiliary nurses *n* = 2 Older adults *n* = 13	Observations Individual interviews	Content analysis	*Three key themes* 1 The location of care 2 The nature of relationship 3 The meaning of ill health and illness
Davies, S Jenkins, E Mabett, G (2010) UK	Qualitative Interpretive	Identify and explore district nurse’s views and perceptions of emotional intelligence	Home care District nurses *n* = 5	Individual interviews	Interpretive phenomeno-logical analysis	*Six themes* 1 Self-awareness 2 Control over emotions 3 Assessment 4 Experience 5 Palliative care 6 Leadership
Annersten, M Pilhammar, K Alm Roijer, C (2012) Sweden	Qualitative Explorative	Explore registered nurse’s professional work with foot ulcer prevention in home care settings	Home care Registered nurses *n* = 15	Individual interviews	Manifest content analysis	*Four themes with subcategories* 1 Leadership: Formal, informal, executive tools 2 Nursing practice: Assessment of patients’ needs, planning, nursing action, evaluation 3 Education: Patient, next of kin, health care assistants, content, educational method 4 Research and development
Berland, A Holm, A Gundersen, D Berntsen, SB (2012) Norway	Qualitative Explorative	Explore home care registered nurses’ experiences of patient safety in the delivery of home care to elderly patients	Home care Registered nurses *n* = 20	Focus groups interviews	Thematic analysis	*One main theme* Struggling with responsibility in different situations: *Five subthemes* 1 Poor work moral and work ethic. 2 Documentation. 3 Lack of functional leadership. 4 Competence. 5 Lack of updated routines and guidelines
Furuåker, C (2012) Sweden	Qualitative Descriptive	Describe the everyday work of registered nurses and their views on what skills they use, require, and wish to develop when looking after patients in home health care	Home care Registered nurses *n* = 20	Individual interviews	Manifest content analysis	*Four themes* 1 Nursing content in home care 2 The home as a workplace 3 Leadership in home care 4 Competence in home care *Five subthemes* 1 Common content. 2 Problematic nursing situations. 3 Required competence. 4 Competence to improve. 5 Learning strategies
Flöjt, J Le Hir, U Rosengren, K (2014) Sweden	Qualitative Descriptive	Describe nurses’ experiences of competence in home health care	Home health care District nurses *n* = 6	Individual interviews	Manifest content analysis	*One category* Being prepared *Three subcategories* 1 Importance of leadership strategies. 2 Training promotes patient safety and independence. 3 Co-operation for professional development
Howell, D Hardy, B Boyd, C et al. (2014) UK	Qualitative Descriptive	Describe community palliative care nurse specialist activities during interaction with patients	Community palliative care Nurse specialist *n* = 4, Older patients *n* = 34	Audio-recorded observations	Thematic analysis	*Summary of interactions* 1 Assessment: History taking, skilled questioning, observation, use of analogue scales, examination 2 Planning: Liaising with others, referral to others, just in case medication, advance care planning 3 Intervention: Clinical, emotional, preparation for death, provision of information, financial, advocacy 4 Evaluation: Follow-up visit or phone call, feedback from others in support team, multidisciplinary team meetings 5 Crosscutting themes: Communication, leadership and coordination, real-time decision making, ability to respond to complex or varied situations
Nilsen, E Olafsen, A Steinsvåg, AG et al. (2016) Norway	Qualitative Descriptive	Present nursing leaders’ role in municipal home care services	Municipal home care services Registered nurses *n* = 9	Individual interviews	Deductive thematic analysis	*Map of interactions and relations between nursing leader and superiors, subordinates and peers* 1 Leadership, managing performance in a squeeze. 2 Relation with the superior, management by email. 3 Relation with subordinates, availability and respect. 4 Relation with peers, professional and personal

### Synthesis of data

The synthesis of *n* = 9 articles was performed by three authors in four stages according to Howell Major and Savin-Baden [16], which originated from meta-ethnography [17]. Stage 1 first-level themes were identified according to the research question and were verified by text citations in several articles (Table 2). Stage 2 s-level themes comprised a reduced form of first-level themes. This was a dynamic process that involved arranging and rearranging the themes several times until clear themes emerged. In Stage 3, patterns and associations between the second-level themes were interpreted, problematized and finally synthesized into an overall third-level theme. This process was repeated until third-level themes were determined. In this process, two themes can be seen to be the same but have different content, which means that there is a need for two separate themes [15, 16]. In Stage 4, an overall assessment was made of the scientific basis and results with 10 themes and conclusions were formulated.

## Results

**Fig. 2:**
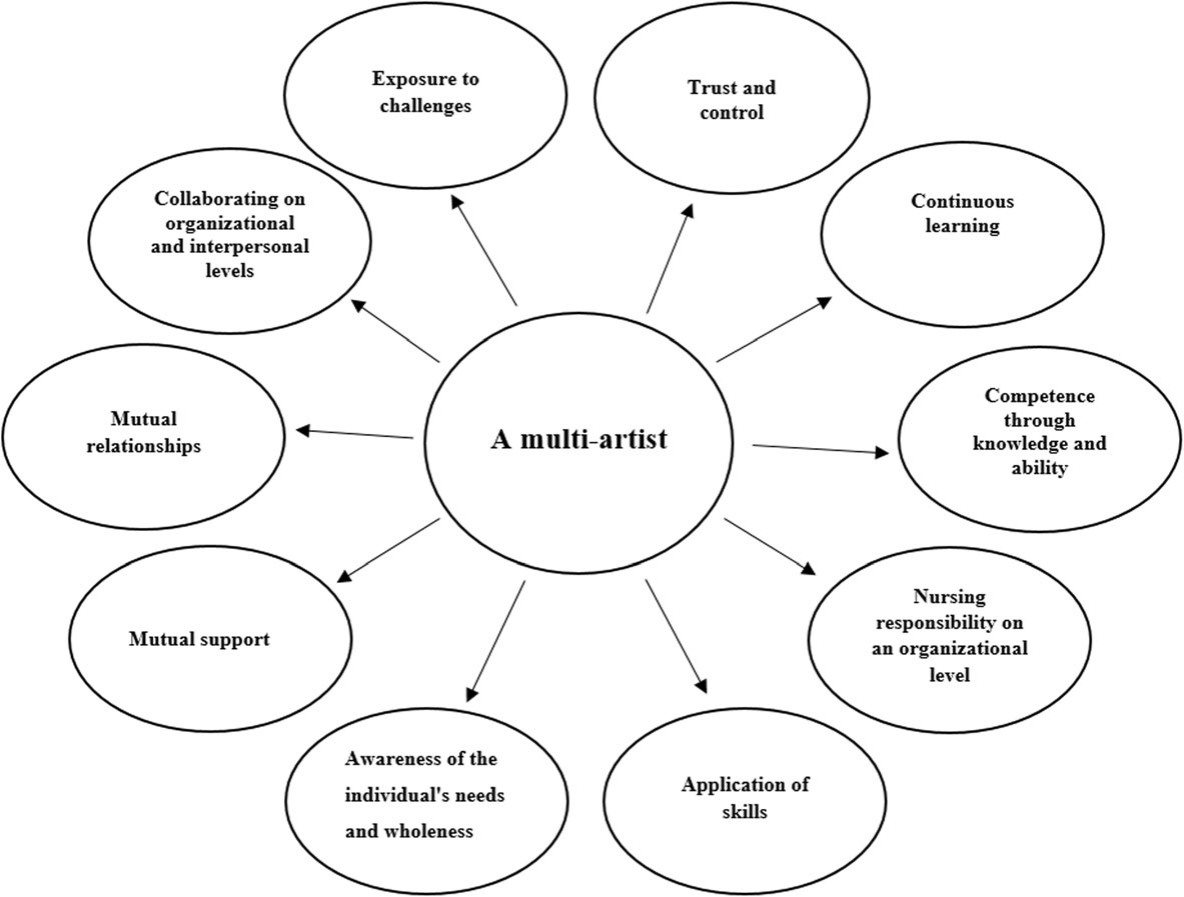
The themes describes registered nurses’ leadership close to older adults in municipal home health care

Through the 10 themes, the results describe what RNs’ leadership implies close to older adults in municipal home health care (Fig. 2).

### Trust and control

RNs’ leadership close to older adults in municipal home health care implies that RNs must be able to trust themselves and the nursing staff and their competence in assessment and information concerning older adults’ condition. RNs must be able to depend on the nursing staffs’ ‘eyes and ears’ and trust all their senses [22–24]. In addition, RNs possess personal characteristics such self-control, which includes RNs’ ability to control and manage their own emotions [25, 26]. RNs must possess self-awareness [25] and be able to set limits for themselves and others [25, 27]. RNs’ leadership implies that they can adapt to older adults’ needs and the wishes of their next of kin [26, 28]. RNs’ leadership also depends on their ability to show their own feelings while simultaneously having the ability to set limits [26]. RNs must also be loyal to nursing staff and management [27].

*… mutual trust was therefore necessary, as the health care assistants constituted the eyes, ears, nose and hands of the registered nurses …* [22], p., 53]

### Continuous learning

RNs’ leadership close to older adults in municipal home health care implies continuous learning in an effort to obtain knowledge in several ways [22, 26]. Learning implies maintaining RNs’ competence, obtaining new knowledge and adapting to new technologies [26, 29]. Learning can be achieved by reflecting on issues with colleagues [27] and through research published in scientific databases and nursing magazines [22]. This can often be performed during RNs’ leisure time [29].

*… I have a number of years of experience and there are always things to learn. So there’s a certain body of knowledge that’s acquired and there’s new things that we learn as we go along …* [26], p., 359]

### Competence through knowledge and ability

Having competence through knowledge and ability means that RNs’ leadership necessitates acquiring knowledge from several different disciplines and contexts, possessing specific medical knowledge and having knowledge in relation to teaching [24]. RNs’ leadership close to older adults involves teaching [22, 26], such as educating nursing staff, older adults and next of kin [22, 24]. Teaching is a positive part of RNs’ leadership roles [24].

*… moreover, teaching knowledge is of great value since teaching is part of leadership, especially with regard to home service enrolled nurses, students, and new colleagues …* [24], p., 225]

*… the registered nurses described how they spent a lot of time and effort educating patients and next of kin, but most of all health care assistants. Most of the patients have concomitant diseases that make education challenging …* [22], p., 55]

Competence through knowledge and ability is also about possessing the capacity to work independently as well as being socially competent and sensitive to others [24, 29].

*… when you work as a nurse in home health care, you make many assumptions. We usually work by ourselves and it’s not always possible to get immediately access to a physician. Then it’s nice to be prepared … I feel comfortable in front of the patient when I know that I have competence …* [29], p., 226]

### Nursing responsibility on an organizational level

RNs’ leadership close to older adults in municipal home health care implies that RNs are responsible on an organizational level. This suggests responsibility for patient safety [23], creating safe and supportive working conditions for nursing staff [27] and evaluating the competence of nursing staff [24]. Moreover, RNs’ leadership means that they are responsible for the care of severely ill older adults [29] and for their individual care plans [23].

*… the nurses expressed concerns over their responsibility for patient safety …* [23], p., 797]

RNs’ nursing responsibility is based on their ability to intervene in situations where patients require care. This includes preventing fall injuries, wound treatment, administering drugs and paying attention to drug side effects [24]. This kind of responsibility also entails the ability to prevent pressure ulcers, contribute to good quality of life in end-of-life care, delegate tasks to nursing staff [22] and prioritize and decide which professionals should be involved in the care [30].

*… the decision making included prioritizing the problems that had been identified, deciding which additional professionals should be involved and when, and making appropriate referrals …* [30], p., 251]

Nursing leadership close to older adults also implies that RNs are responsible for documenting nursing interventions according to existing legislation [22, 24] and evaluating the performance of nursing interventions [24, 30].

*… after the first visit RN’s prepare an individual care plan, which is carefully documented, and this plan is then supposed to be followed up and implemented by EN’s. RN’s continuously document each visit, telephone contact with EN’s, doctors, rehabilitation staff, and next of kin …* [24], p., 223]

### Application of skills

The application of skills means that RNs have experience and apply it in their work [26]. RNs often have experience in different kinds of disciplines and contexts [24]. Using their skills also implies that they employ all their senses in nursing assessments [30] and be prepared to use their intuition and abilities [24, 25, 29].

*… the assessment process involved use of several techniques, including detailed close, open, and probing questioning; observation (*e.g. *of mobility, wellbeing, and anxiety); physical examination (*e.g. *of syringe driver sites, oral hygiene, and abdominal distension using palpation); and use of measurement tools (including analogue scales to measure pain and response to analgesia) …* [30], p., 248]

### Awareness of the individual’s needs and wholeness

RNs’ leadership close to older adults implies their ability to determine the individual’s needs and be aware regarding the notion wholeness. Awareness of the individual’s needs means that RNs must be able to intuit such needs by ‘hearing’ words that are not spoken [25]. Sometimes this involves simply being there for the patient [24]. Moreover, an awareness of individuals’ needs also necessitates that RNs perform medical assessments [22, 24] as well as encourage older adults to participate in their own self-care [28]. RN’s leadership close to older adults always implies addressing older adults’ needs in a holistic manner [22, 24].

*… a holistic view of the patients was emphasized, also for non-ulcerated patients with diabetes …* [22], p., 55]

*… a person at home becomes more individual … you take care of a whole human being …* [24], p., 224]

### Mutual support

Mutual support shows that the RNs both give and receive support from colleagues. This could include sharing one’s knowledge, supporting each other and receiving support [26, 27]. Asking colleagues for help in both small and larger matters seems to be an important way of engendering a supportive work environment [27].

*… and if I spoke to a colleague, with the experience she has, I would have said to her, This and this is happening, then she would tell me; look, try this to see what it could do, and if it doesn’t work call me back …* [26], p., 359]

### Mutual relationships

Mutual relationships assumes that RNs perform their tasks professionally in relation to colleagues [27], older adults and next of kin. It also implies creating good relationships with older adults and next of kin by providing support, understanding and reassurance [26, 28, 30]. Mutual relationships also include dealing with communication problems using different communication techniques [24, 30].

*… nurses described how first assessment was regarded as a way of establishing and developing relationships between nurse and patient. As such, nurses described how they viewed first assessment as enabling them to establish the bonds or sow the seeds that would nurture or grow future relationships …* [28], p., 86]

### Collaborating on organizational and interpersonal levels

Collaborating on an organizational level is about teamwork within the organization. It can involve teamwork with the RNs’ manager, colleagues, subordinates or other professionals and with older adults and next of kin [24, 26, 27, 29, 30]. Collaborating on an interpersonal level, alternatively, involves working in partnership with care units where the RNs act as a link between older adults and their next of kin [26, 30].

*… we make suggestions to each other, and I say, ‘well what do you think about this? ‘or they might say, ‘what do you think about that? ‘So we’re kind of collaborating as team members …* [26], p., 360]

*… this was particularly apparent in relation to the provision of home health care, where they often provided the link between primary, secondary, and tertiary care as well as with other agencies, such as hospices …* [30], p., 250]

### Exposure to challenges

RNs are exposed to different kinds of challenges, such as handling problematic situations [24], confronting and managing complex demands and contradictory feelings from older adults and next of kin [26, 27] and dealing with obstacles posed by different education situations [22]. RNs are also exposed to challenges related to deficiencies in appropriate routines, feeling forced to compromise on patient safety in some situations [23] and feeling powerless [27].

*… it was also difficult for them to deal with family members’ conflicting points of views, for instance, in the case where some children believe their parent should be left to die peacefully and the other ones believe in giving hydration or intervention at any cost …* [26], p., 360]

## Discussion

RNs as leaders implies being multi-artists (Fig. 2), that is, possessing good self-knowledge combined with knowledge and experience of the role attributed to RNs’ ability to practice leadership close to older adults for the benefit of older adults and their next of kin [25, 27]. RNs need to have confidence to be able to trust nursing staff and their performance as well as nursing staff’s ability to adapt to the needs of older adults [22, 24]. The ability to control and manage one’s emotions is crucial to RNs’ ability not to take difficult situations personally and to maintain their commitment to older adults and their next of kin in a professional manner [26]. RNs also have to demonstrate loyalty to both top management and the nearest management as well as to colleagues and nursing staff. This proves to be an important way that RNs contribute to fostering positive relationships in the development of good care for older adults [27]. Heffernan and Quinn Griffin [31] confirm that there is a positive correlation between RNs’ self-esteem and emotional intelligence in the way that they impact RNs’ ability to show compassion, which forms an important part of nursing care. Durkin and Beaumont [32] confirm that RNs’ high levels of self-compassion in the context of municipal home health care could be linked to lower levels of burnout and also shows that RNs could feel more sympathy towards others [32]. In relation to the development of good health care provided for older adults, this highlights that RNs’ personalities are an important part of their leadership skills in working closely with older adults in municipal home health care.

It is also implied that RNs require consistent learning and development in order to preserve and expand their own competence [22, 26]. RNs learn by reflecting on issues with colleagues [27]. However, ongoing learning also means that they continue to study in their field during leisure time [24]. Earlier research [12] confirmed that education levels, age and years of experience have an impact on RNs’ self-rated competence. Moreover, tailored education is crucial in order for RNs to comply with the necessary requirements to provide effective care for older adults [12]. This also applies to evidence-based nursing, which is one of the six core competencies in nursing [33].

This study pointed out that RNs are dependent on the experiences gained from different kinds of disciplines and contexts [24]. It also showed that the ability to use lessons learned from these experiences was important [26]. The results showed that using senses, intuition and abilities in the assessment process was imperative [30]. Penney and Poulter [34] confirm that one part of RNs’ nursing assessment relates to an unspoken assessment of prior experience as well as RNs’ intuition. RNs interpreted this silent assessment process as a subconscious action [34]. This means that the assessment process is a complex situation where RNs’ leadership requires that they use their whole being and all their senses in combination with professional competence and working skills.

RNs’ leadership close to older adults in municipal home health care suggests a need for teaching ability [24] and the ability to work independently [24, 29]. Teaching includes informing, educating and teaching nursing staff, older adults and next of kin [22, 24, 26]. Teaching is a positive part of RNs’ leadership performance [24] even though it takes away a significant amount of time from other duties and can be very challenging [22]. Blevins [35] confirms that educating patients is an ongoing process that facilitates patients’ involvement in planning and handling. For education to be effective, RNs must ensure they meet patients’ specific needs [35]. Therefore, it is important that RNs are given time for the education process. Bing-Jonsson and Bjork [36] show a variety of competencies that RNs working in home health care need in order to prevent diseases and care for older adults in their home [36]. RNs’ leadership thus implies that they need personal experience and competence in several disciplines in order to feel comfortable in their professional role and to contribute to safe care.

RNs’ leadership also suggests organizational responsibility for nursing care and patient safety [23] as well as the ability to create prerequisites for the work performed by nursing staff [27]. This implies a need to evaluate the nursing staff’s duties and the individual care plans for older adults [22]. RNs’ organizational responsibility also indicates a need to prioritize and decide which professionals should be involved in the care of older adults [30]. Reaearch [37] confirms that RNs’ confidence in delegating assignments and duties aligns with the duration of their total nursing experiences, delegation-training experiences and experiences in leadership. This is in line with earlier research [38] showing that evaluation and strong communication between RNs and nursing staff are important factors in maintaining patient safety when responsibilities are delegated. This is in line with patient safety, a core competence in nursing, with the goal to minimize the risk of harm to patients and nursing staff through system effectiveness and individual performance [33]. However, this poses a considerable challenge for RNs in their ability to perform their leadership duties because it takes years to develop experience and become confident in managing all of these responsibilities.

The ability to understand individuals’ needs from a holistic view refers to the fact that RNs’ leadership close to older adults in municipal home health care requires that they be able to intuit their patients’ needs [25] and provide holistic care [22, 24]. RNs also have a responsibility to encourage older adults to participate in their own self-care [28]. Earlier research [39] confirmed that RNs who had a positive attitude were better able to encourage patients to participate in their own care and that this was crucial for older adults. Conversely, a negative attitude could create a barrier for patient participation. Fagerberg and Engström [40] confirm that RNs’ knowledge about older adults’ earlier lives gives RNs satisfaction and enables them to create a close bond with older adults. However, this relationship could sometimes be demanding, especially if the older adults feel their lives should be more private.

The results clarified that RNs both gave and received support to and from colleagues. This included sharing knowledge with each other [26, 27]. These positive relations are considered to be an important part of RNs’ work environment [27]. Earlier research [41] confirmed that commitment and competence are crucial to positive leadership.

The results clarified that RNs’ leadership was characterized by mutual relations based on relationships with colleges, older adults and their next of kin. These relationships were based on RNs’ professional attitude and ability to listen to, support and reassure patients [26, 28, 30]. Marshall [42] confirms that RNs’ attitude towards older adults is crucial to the quality of care received, where a positive attitude towards older adults is a precondition for a good care [42]. Building a faithful and trustworthy relationship with older adults and their next of kin is certainly one of the largest challenges RNs face. The challenge is to create a positive caring relationship with the older adult [43], a relationship that becomes the foundation for all professional interactions [43].

RNs’ leadership close to older adults in municipal home health care implies a need to collaborate on an organizational level, a requirement that is grounded in RNs’ involvement with the internal organization. In the care situation, the composition of team members could vary among subordinate staff, colleagues, other care professionals and also between, and with, older adults and their next of kin [24, 26, 27, 29, 30]. Dahlke and Stahlke [44] define the impact of positive team relationships on health care workers’ job satisfaction. They also identified how these relationships affected the care of older adults, such as the fact that when health care workers worked collaboratively, their efforts positively benefited the older adults in their care [44]. Because of RNs’ unique role in being the people who have a holistic view of older adults’ needs, their leadership close to older adults in municipal home health care seems to necessitate collaboration on an organizational level. This highlights RNs’ leadership close to older adults as important and crucial in the design of health care tailored to older adults’ implicit and explicit needs. Alternatively, interpersonal collaboration between care units is important because RNs act as a link between these units, older adults and their next of kin [26, 30]. The aging population requires care, and this translates into vulnerability. RNs’ leadership close to older adults in municipal home health care is an important part of the health care process; in part it supports and caters to older adults’ needs for nursing and care, and it can also be a means of using health care resources as wisely and sustainably as possible. This is in line with core competencies in nursing through the competencies of teamwork, collaboration and person-centred care [33].

RNs’ leadership close to older adults’ in municipal home health care may also mean that they face problematic situations in direct nursing care [24], such as managing older adults’ emotional reactions and complex requirements [26, 27] as well as facing challenges in educational situations [22]. Exposure to challenges also suggests that, in some cases, RNs’ work suffers from lack of routine. Due to the lack of routines, RNs have concerns about patient safety, which in some cases meant that RNs were forced to compromise on patient safety [23]. RNs might also experience a sense of powerlessness because of shortcomings in the organization [27].

Åhlin [45] pointed out that nursing staff need attentive and supportive leaders who help them prioritize the daily care of older adults. This seems to be important whether the priorities relate to guidelines and older adults’ needs or arise as a consequence of a stressful work environment [45]. RNs’ leadership close to older adults needs to be given the appropriate conditions that will allow them to cope with the challenges they face on a daily basis in order to meet older adults’ individual and specific needs and for the implementation of safe care.

### Study limitations

A systematic review of qualitative research indicated that there could be weaknesses in the search strategy, especially when there are discrepancies in the database indexing [15]. In this study, the search strategy was performed in conjunction with an information specialist. Two independent researchers in the field also audited the search strategy. Search themes were carefully selected according to inclusion criteria related to the research question. The choice of two databases may have been a weakness; however, the chosen databases did cover the research field. The choice to exclude and include abstracts might have been a weak one. However, in this study, three researchers blindly selected articles in the web application Rayyan QCRI [19]. Moreover, the research group held frequent seminars to illuminate the details in every step of the search and analysis process as well as in the quality assessment in order to adhere to data quality and prevent biases. Although the results of this study relate to a special context in RN s’ leadership close to older adults in municipal home health care, there may be similarities to RNs’ leadership in other contexts.

## Conclusions

RNs as leaders close to older adults in municipal home health care are suggested to be multi-artists. Nursing education, including specialist education for RNs, should prepare them for their unique and complex leadership role as multi-artists. Municipal employers require knowledge about what RNs’ leadership implies in order to create adequate conditions for their roles in achieving safe care. Further research is warranted to explore RNs’ leadership close to older adults in municipal home health care from different perspectives, such as older adults and next of kin.

## Data Availability

The dataset from this study is available from the corresponding author upon reasonable request.

## Abbreviation

RN: Registered nurse

## References

[1] Josefsson K, Hansson M. To lead and to be led in municipal elderly care in Sweden as perceived by registered nurses. J Nurs Manag. 2011. 10.1111/j.1365-2834.2011.01228.x. 10.1111/j.1365-2834.2011.01228.x 21569146

[2] Sullivan EJ, Garland G (2010). Practical leadership and management in nursing.

[3] Avolio BJ, Walumbwa FO, Weber TJ. Leadership: current theories, research, and future directions. Annu Rev Psychol. 2009. 10.1146/annurev.psych.60.110707.163621. 10.1146/annurev.psych.60.110707.163621 18651820

[4] Arman M, Ranheim A, Rydenlund K, Rytterström P, Rehnsfeldt A. The Nordic tradition of caring science: the works of three theorists. Nurs Sci Q. 2015. 10.1177/0894318415599220. 10.1177/0894318415599220 26396212

[5] Arman M, Dahlberg K, Ekebergh M (2015). Teoretiska grunder för vårdande.

[6] International council of nurses (2012). The ICN code of ethics for nurses.

[7] SSF (2017). Kompetensbeskrivning för legitimerad sjuksköterska [Competency description for registered nurses].

[8] Stanley D, Stanley K. Clinical leadership and nursing explored A literature search. J Clin Nurs. 2018. 10.1111/jocn.14145. 10.1111/jocn.14145 29076264

[9] Coster S, Watkins M, Norman IJ. What is the impact of professional nursing on patients’ outcomes globally? An overview of research evidence. Int J Nurs Stud. 2018. 10.1016/j.ijnurstu.2017.10.009. 10.1016/j.ijnurstu.2017.10.009 29110907

[10] Ljungbeck B, Sjögren FK. Advanced nurse practitioners in municipal healthcare as a way to meet the growing healthcare needs of the frail elderly: a qualitative interview study with managers, doctors and specialist nurses. BMC Nurs. 2017. 10.1186/s12912-017-0258-7. 10.1186/s12912-017-0258-7 PMC5689167 29176932

[11] Josefsson K (2006). Municipal elderly care: implications of registered nurses’ work situation, education, and competence. [dissertation].

[12] Karlstedt M, Wadensten B, Fagerberg I, Pöder U. Is the competence of Swedish registered nurses working in municipal care of older people merely a question of age and postgraduate education? Scand J Caring Sci. 2015. 10.1111/scs.12164. 10.1111/scs.12164 25213399

[13] WHO (2015). The growing need for home health care for the elderly: Home health care for the elderly as an integral part of primary health care services. Regional Office for the Eastern Mediterranean: World Health Organization.

[14] Moher D, Liberati A, Tetzlaff J, Altman DG. Preferred reporting items for systematic reviews and meta-analyses: the PRISMA statement. PLoS Med. 2009. 10.1371/journal.pmed.1000097. PMC3090117 21603045

[15] SBU (2016). Evaluation and synthesis of studies using qualitative methods of analysis.

[16] Howell Major C, Savin-Baden M (2010). An introduction to qualitative research synthesis: managing the information explosion in social science research.

[17] Noblit GW, Hare RD (1988). Meta-ethnography synthesizing qualitative studies.

[18] Curtis EA, de Vries J, Sheerin FK. Developing leadership in nursing: Exploring core factors. Br J Nurs. 2011. 10.12968/bjon.2011.20.5.306. 10.12968/bjon.2011.20.5.306 21471879

[19] Ouzzani M, Hammady H, Fedorowicz Z, Elmagarmid A. Rayyan—a web and mobile app for systematic reviews. Systematic Reviews. 2016. 10.1186/s13643-016-0384-4. 10.1186/s13643-016-0384-4 PMC5139140 27919275

[20] The Joanna Briggs institute critical appraisal tools for use in JBI systematic reviews. https://joannabriggs.org/ebp/critical_appraisal_tools. Accessed 8 Jan 2020.

[21] Lockwood C, Munn Z, Porritt K. Qualitative research synthesis: methodological guidance for systematic reviewers utilizing meta-aggregation. Int J Evid Based Healthc. 2015. 10.1097/xeb.0000000000000062. 10.1097/XEB.0000000000000062 26262565

[22] Annersten Gershater M, Pilhammar E, Alm RC. Prevention of foot ulcers in patients with diabetes in home nursing: a qualitative interview study. Eur Diabetes Nurs. 2013. 10.1002/edn.227.

[23] Berland A, Holm AL, Gundersen D, Bentsen SB. Patient safety culture in home care: experiences of home-care nurses. J Nurs Manag. 2012. 10.1111/j.1365-2834.2012.01461.x. 10.1111/j.1365-2834.2012.01461.x 22967297

[24] Furåker C. Registered nurses’ views on competencies in home care. J Nurs Manag. 2012. 10.1177/1084822312439579. 19094105

[25] Davies S, Jenkins E, Mabbett G (2010). Emotional intelligence: district nurses’ lived experiences. Br J Community Nurs.

[26] Arnaert A, Wainwright M. Providing care and sharing expertise: reflections of nurse-specialists in palliative home care. Palliat Support Care. 2009. 10.1017/s1478951509990290. 10.1017/S1478951509990290 19788778

[27] Nilsen ER, Olafsen AH, Steinsvag AG, Halvari H, Grov EK (2016). Stuck between a rock and a hard place: the work situation for nurses as leaders in municipal health care. J Multidiscip Healthc.

[28] McGarry J. Defining roles, relationships, boundaries and participation between elderly people and nurses within the home: an ethnographic study. Health Soc Care Community. 2009. 10.1111/j.1365-2524.2008.00802.x. 10.1111/j.1365-2524.2008.00802.x 18700870

[29] Flöjt J, Hir UL, Rosengren K. Need for preparedness: nurses’ experiences of competence in home health care. Home Health Care Manag Pract. 2014. 10.1177/1084822314527967.

[30] Howell D, Hardy B, Boyd C, Ward C, Roman E, Johnson M. Community palliative care clinical nurse specialists: A descriptive study of nurse-patient interactions. Int J Palliat Nurs. 2014. 10.12968/ijpn.2014.20.5.246. 10.12968/ijpn.2014.20.5.246 24852032

[31] Heffernan M, Quinn Griffin MT, McNulty SR, Fitzpatrick JJ. Self-compassion and emotional intelligence in nurses. Int J Nurs Pract. 2010. 10.1111/j.1440-172X.2010.01853.x. 10.1111/j.1440-172X.2010.01853.x 20649668

[32] Durkin M, Beaumont E, Hollins Martin CJ, Carson J. A pilot study exploring the relationship between self-compassion, self-judgement, self-kindness, compassion, professional quality of life and wellbeing among UK community nurses. Nurse Educ Today. 2016. 10.1016/j.nedt.2016.08.030. 10.1016/j.nedt.2016.08.030 27621200

[33] Cronenwett L, Sherwood G, Barnsteiner J, Disch J, Johnson J, Mitchell P, Sullivan DT, Warren J. Quality and safety education for nurses. Nurs Outlook. 2007. 10.1016/j.outlook.2007.02.006. 10.1016/j.outlook.2007.02.006 17524799

[34] Penney W, Poulter N, Cole C, Wellard S. Nursing assessment of older people who are in hospital: exploring registered nurses’ understanding of their assessment skills. Contemp Nurse. 2016. 10.1080/10376178.2015.1111152. 10.1080/10376178.2015.1111152 26493741

[35] Blevins S (2018). The art of patient education. Medsurg Nurs.

[36] Bing-Jonsson PC, Bjork IT, Hofoss D, Kirkevold M, Foss C. Competence in advanced older people nursing: development of ‘nursing older people--competence evaluation tool’. Int J Older People Nursing. 2015. 10.1111/opn.12057. 10.1111/opn.12057 24863394

[37] Yoon J, Kim M, Shin J. Confidence in delegation and leadership of registered nurses in long-term-care hospitals. J Nurs Manag. 2016. 10.1111/jonm.12372. 10.1111/jonm.12372 27029905

[38] Gransjön Craftman Å, Grape C, Ringnell K, Westerbotn M. Registered nurses' experience of delegating the administration of medicine to unlicensed personnel in residential care homes. J Clin Nurs. 2016. 10.1111/jocn.13335. 10.1111/jocn.13335 27461555

[39] Larsson IE, Sahlsten MJM, Segesten K, Plos KAE. Patients’ perceptions of barriers for participation in nursing care. Scand J Caring Sci. 2011. 10.1111/j.1471-6712.2010.00866.x. 10.1111/j.1471-6712.2010.00866.x 21241347

[40] Fagerberg I, Engström G. Care of the old—a matter of ethics, organization and relationships. Int J Qual Stud Health Well-Being. 2012. 10.3402/qhw.v7i0.9684. 10.3402/qhw.v7i0.9684 PMC3349146 22577469

[41] Lunden A, Teräs M, Kvist T, Häggman-Laitila A. A systematic review of factors influencing knowledge management and the nurse leaders’ role. J Nurs Manag. 2017. 10.1111/jonm.12478. 10.1111/jonm.12478 28580645

[42] Marshall L (2010). Potential implications of registered nurse attitudes towards caring for older people. Contemp Nurse.

[43] Todres L, Galvin K, Dahlberg K. Lifeworld-led healthcare: revisiting a humanising philosophy that integrates emerging trends. Med Healthcare Philos. 2007. 10.1007/s11019-006-9012-8. 10.1007/s11019-006-9012-8 16847724

[44] Dahlke S, Stahlke S, Coatsworth-Puspoky R. Influence of teamwork on health care workers’ perceptions about care delivery and job satisfaction. J Gerontol Nurs. 2018. 10.3928/00989134-20180111-01. 10.3928/00989134-20180111-01 29355879

[45] Åhlin J (2015). Stress of conscience and burnout among healthcare personnel working in residential care of older people [dissertation].

